# Observations of infection prevention and control practices in primary health care, Kenya

**DOI:** 10.2471/BLT.16.179499

**Published:** 2017-03-09

**Authors:** Guadalupe Bedoya, Amy Dolinger, Khama Rogo, Njeri Mwaura, Francis Wafula, Jorge Coarasa, Ana Goicoechea, Jishnu Das

**Affiliations:** a Development Economics Research Group, The World Bank, 1818 H St NW, Washington DC, 20433, United States of America (USA).; b Health, Nutrition and Population Global Practice, The World Bank, Nairobi, Kenya.; c Health, Nutrition and Population Global Practice, The World Bank, New Delhi, India.; d Trade and Competitiveness Global Practice, The World Bank, Washington DC, USA.

## Abstract

**Objective:**

To assess compliance with infection prevention and control practices in primary health care in Kenya.

**Methods:**

We used an observational, patient-tracking tool to assess compliance with infection prevention and control practices by 1680 health-care workers during outpatient interactions with 14 328 patients at 935 health-care facilities in 2015. Compliance was assessed in five domains: hand hygiene; protective glove use; injections and blood sampling; disinfection of reusable equipment; and waste segregation. We calculated compliance by dividing the number of correct actions performed by the number of indications and evaluated associations between compliance and the health-care worker’s and facility’s characteristics.

**Findings:**

Across 106 464 observed indications for an infection prevention and control practice, the mean compliance was 0.318 (95% confidence interval, CI: 0.315 to 0.321). The compliance ranged from 0.023 (95% CI: 0.021 to 0.024) for hand hygiene to 0.871 (95% CI: 0.866 to 0.876) for injection and blood sampling safety. Compliance was weakly associated with the facility’s characteristics (e.g. public or private, or level of specialization) and the health-care worker’s knowledge of, and training in, infection prevention and control practices.

**Conclusion:**

The observational tool was effective for assessing compliance with infection prevention and control practices across multiple domains in primary health care in a low-income country. Compliance varied widely across infection prevention and control domains. The weak associations observed between compliance and the characteristics of health-care workers and facilities, such as knowledge and the availability of supplies, suggest that a broader focus on behavioural change is required.

## Résumé

**Objectif:**

Évaluer le respect des pratiques de prévention et de lutte contre les infections lors d’activités de soins primaires au Kenya.

**Méthodes:**

Grâce à un outil observationnel de suivi des patients, nous avons évalué le respect des pratiques de prévention et de lutte contre les infections par 1 680 agents de santé, lors de leurs interactions avec 14 328 patients, pris en charge en consultation externe dans 935 centres de santé en 2015. Le respect de ces pratiques a été évalué dans cinq domaines: hygiène des mains; utilisation de gants de protection; injections et prélèvements sanguins; désinfection des équipements réutilisables et tri des déchets. Nous avons calculé le niveau de respect de ces pratiques en divisant le nombre d'actions correctes par le nombre de cas pour lesquels l'application de ces pratiques était indiquée, et nous avons évalué les liens éventuels entre le respect des bonnes pratiques et les caractéristiques des agents de santé et des centres de santé.

**Résultats:**

Sur les 106 464 cas observés pour lesquels l'application des pratiques de prévention et de lutte contre les infections était indiquée, le respect de ces pratiques a été évalué à un niveau moyen de 0,318 (intervalle de confiance de 95%, IC: 0,315 à 0,321). Le niveau de respect a varié entre 0,023 (IC 95%: 0,021 à 0,024) pour l'hygiène des mains et 0,871 (IC 95%: 0,866 à 0,876) pour la sûreté des injections et des prélèvements. Le niveau de respect de ces pratiques semble peu dépendre des caractéristiques du centre de santé (ex. : établissement public, privé ou niveau de spécialisation) ou des connaissances des pratiques de prévention et de lutte contre les infections par les agents de santé et de leur formation à ces pratiques.

**Conclusion:**

L'outil observationnel s'est avéré efficace pour évaluer le niveau de respect des pratiques de prévention et de lutte contre les infections, dans de nombreux domaines liés aux soins de santé primaires, dans un contexte de faibles ressources. Le niveau de respect varie considérablement en fonction du domaine de pratiques considéré. Le lien ténu observé entre le niveau de respect et les caractéristiques des agents de santé ou des centres de santé (connaissances des agents ou disponibilité des fournitures nécessaires, par exemple) suggère que l'accent doit encore être davantage mis sur le changement des comportements.****

## Resumen

**Objetivo:**

Evaluar el cumplimiento de las prácticas de prevención y control de infecciones en la atención sanitaria primaria en Kenya.

**Métodos:**

Se utilizó una herramienta de observación para el seguimiento de los pacientes con el fin de evaluar el cumplimiento de las prácticas de prevención y control de infecciones por parte de 1 680 trabajadores sanitarios durante interacciones ambulatorias con 14 328 pacientes en 935 centros sanitarios en 2015. El cumplimiento se evaluó en cinco ámbitos: higiene de las manos, uso de guantes protectores, inyecciones y muestras de sangre, desinfección de equipos reutilizables y separación de residuos. Se calculó el cumplimiento dividiendo el número de acciones correctas realizadas entre el número de indicaciones y asociaciones evaluadas entre el cumplimiento, los trabajadores sanitarios y las características de los centros.

**Resultados:**

En 106 464 indicaciones observadas para las prácticas de prevención y control de infecciones, el cumplimiento medio fue de un 0,318 (intervalo de confianza, IC, del 95%: 0,315 a 0,321). El cumplimiento varió de un 0,023 (IC del 95%: 0,021 a 0,024) para la higiene de las manos a un 0,871 (IC del 95%: 0,866 a 0,876) para la seguridad de las inyecciones y las muestras de sangre. El cumplimiento apenas se relacionó con las características del centro (por ejemplo, público o privado, o nivel de especialización) y el conocimiento y la formación del trabajador sanitario en cuanto a prácticas de prevención y control de infecciones.

**Conclusión:**

La herramienta de observación fue eficaz para evaluar el cumplimiento de las prácticas de prevención y control de infecciones en varios ámbitos de la atención sanitaria primaria en un país de ingresos bajos. El cumplimiento varió enormemente en los ámbitos de prevención y control de infecciones. Las escasas asociaciones observadas entre el cumplimiento y las características de los trabajadores sanitarios y los centros, como los conocimientos y la disponibilidad de suministros, sugieren que es necesario un mayor enfoque sobre el cambio de comportamiento.

## ملخص

**الغرض:**

تقييم الامتثال لعمليات ممارسة مكافحة العدوى والوقاية منها في الرعاية الصحية الأولية في كينيا.

**الطريقة:**

استخدمنا أداة رصدية وتتبعية للمريض لتقييم امتثاله لعمليات ممارسة مكافحة العدوى والوقاية منها من قبل 1680 من العاملين في مجال الرعاية الصحية أثناء التفاعلات الخارجية مع 14,328 مريض في 953 منشأة رعاية صحية في عام 2015. وتم تقييم الامتثال على خمسة محاور: نظافة اليد، واستخدام القفازات الواقية، وعينات الدم والحقن، وتعقيم المواد التي يُعاد استخدامها، وفرز النفايات. وقمنا بحساب الامتثال عن طريق قسمة عدد الإجراءات الصحية التي تم اتخاذها على عدد المؤشرات والجمعيات التي تم تقييمها وفقًا للامتثال والعاملين بالرعاية الصحية وخصائص المنشآت الصحية.

**النتائج:**

عبر 106,464 مؤشر ملحوظ لعملية ممارسة مكافحة العدوى والوقاية منها، كان متوسط الامتثال 0.318 (بنسبة أرجحية مقدارها 95‏%: 0.315 إلى 0.321). وتراوح الامتثال لنظافة اليد ما بين 0.023 (بنسبة أرجحية مقدارها ‏‏95%: 0.021 إلى 0.024) إلى 0.871 (بنسبة أرجحية مقدارها 95‏%: 0.866 إلى 0.876) لسلامة عينات الدم والحقن. ويرتبط الامتثال بشكل ضعيف مع خصائص المنشأة الطبية (على سبيل المثال ما إذا كانت عامة أو خاصة، أو مستوى التخصص) ومعرفة عامل الرعاية الصحية بعمليات ممارسة مكافحة العدوى والوقاية منها وتدريبه على تلك العمليات.

**الاستنتاج:**

كانت الأداة الرصدية فعالة في تقييم الامتثال لعمليات ممارسة مكافحة العدوى والوقاية منها عبر محاور متعددة في الرعاية الصحية الأولية في دولة منخفضة الدخل. وقد اختلف الامتثال بشكل واسع عبر محاور مكافحة العدوى والوقاية منها. وتشير الارتباطات الضعيفة الملحوظة بين الامتثال وخصائص العاملين في الرعاية الصحية والمنشآت الصحية – مثل المعرفة وتوفر المواد – إلى ضرورة وجود تركيز أوسع على التغييرات السلوكية.

## 摘要

**目的:**

旨在评估肯尼亚初级卫生护理中感染防控做法的合规性。

**方法:**

2015 年，我们采用观察性患者追踪工具对 935 家卫生护理机构 1680 名卫生保健工作人员在 14 328 名患者门诊会谈中的感染防控做法的合规性进行了评估。我们对五个领域的合规性进行了评估： 手卫生；防护手套的使用；注射和血液样本；可重复使用设备的消毒以及废弃物隔离。 我们通过正确行为数目除以指标数目计算了合规性，同时评估了合规性与卫生护理工作人员和机构特点之间的关联。

**结果:**

在一项感染防控做法的 106 464 个观察指标中，合规率为 0.318（95% 置信区间，CI： 0.315 至 0.321）。 合规率范围包括从手卫生的 0.023（95% CI： 0.021 至 0.024）到感染和血液样本安全性的 0.871（95% CI： 0.866 至 0.876）。 合规性与机构的特点（例如公立或私立，或专业化程度）、卫生护理工作人员有关感染防控做法的知识以及在此方面的培训关联性不大。

**结论:**

观察工具在评估低收入国家多个初级卫生护理领域的感染防控做法的合规性方面卓有成效。 感染防控领域之间的合规性差别很大。 据观察，合规性与卫生护理工作人员和机构的特点（如知识和物资供应）的关联性不大，这表明需要加大对行为变化的关注。

## Резюме

**Цель:**

Провести оценку степени соблюдения мероприятий по профилактике и контролю инфекций в первичной медико-санитарной помощи в Кении.

**Методы:**

Мы использовали инструмент для отслеживания пациентов для оценки степени соблюдения мероприятий по профилактике и контролю инфекций 1680 работниками здравоохранения во время амбулаторного приема 14 328 пациентов в 935 медицинских учреждениях в 2015 году. Степень соблюдения оценивали в пяти областях: гигиена рук, использование защитных перчаток, инъекции и забор крови, дезинфекция многоразового оборудования, сортировка отходов. Мы рассчитали степень соблюдения путем деления количества выполненных правильных действий на количество показаний и провели оценку взаимосвязи между соблюдением и характеристиками работников здравоохранения и медицинских учреждений.

**Результаты:**

В 106 464 наблюдаемых показаниях для мероприятий по профилактике и контролю инфекций среднее значение степени соблюдения составило 0,318 (95%-й доверительный интервал, ДИ: от 0,315 до 0,321). Значения степени соблюдения варьировались от 0,023 (95%-й ДИ: от 0,021 до 0,024) для гигиены рук до 0,871 (95%-й ДИ: от 0,866 до 0,876) для безопасного проведения инъекций и забора крови. Степень соблюдения была слабо связана с характеристиками медицинских учреждений (например, государственные или частные, уровень специализации) и квалификацией работников здравоохранения, а также с обучением по проведению мероприятий по профилактике и контролю инфекций.

**Вывод:**

Инструмент наблюдения был эффективен для оценки степени соблюдения мероприятий по профилактике и контролю инфекций в нескольких областях в первичной медико-санитарной помощи в стране с низким уровнем дохода. Степень соблюдения широко варьируется по областям применения профилактики и контроля инфекций. Слабая взаимосвязь, наблюдаемая между степенью соблюдения и характеристиками работников здравоохранения и медицинских учреждений, таких как квалификация и доступность расходных материалов, позволяет предположить, что требуется обратить более пристальное внимание на изменение поведения.****

## Introduction

The prevention and control of infections is critical for a well-functioning health system. However, worldwide an estimated 21 million cases of hepatitis B virus infection and 200 000 cases of human immunodeficiency virus (HIV) infection result from unsafe injection practices each year.^1^ In the United States of America, an estimated 40 000 to 80 000 deaths are due to nosocomial infections annually, which may cost as much as 4.5 billion United States dollars (US$).^2^ Moreover, the rapid spread of multidrug-resistant organisms and outbreaks of Ebola virus disease, yellow fever and Zika virus infections has further increased the human and financial cost. Fortunately, proven and cost-effective, infection prevention and control practices can reduce the risk.^3^^–^^6^

However, ensuring compliance with these practices depends on understanding the extent of the problem and there has been little research on infection prevention and control practices in low- and middle-income countries, particularly in primary health care.^7^^–^^10^ Previous studies have several limitations. First, many involved small samples – one review found that only 10 of 41 studies on hand hygiene interventions were conducted in more than one hospital.^10^^,^^11^ Second, they were often based on self-reported data from health-care providers, which tend to overestimate compliance.^12^ Third, they frequently focused on single domains, such as injection safety, rather than on the range of possible exposures encountered by patients during outpatient visits.^1^^,^^13^^–^^17^ Consequently, given that infections are transmitted by multiple pathways, these data are of limited use for modelling the spread of different pathogens.^10^^,^^18^

To address these limitations, we designed a novel observational tool. We based the tool on existing World Health Organization (WHO) tools that can track patients throughout the course of an outpatient visit, which may include examinations, laboratory tests and injections. Our tool can be used to assess compliance with infection prevention and control practices throughout primary health care and can help identify associations between compliance and individual characteristics of health-care facilities and workers.

We used the observational tool to investigate compliance with infection prevention and control practices in a pilot sample of 23 health-care facilities in Nairobi, Kenya and, subsequently, in 935 facilities in three Kenyan counties. 

## Methods

The design of the observational tool took into account the possibility that an outpatient may experience a violation of infection prevention and control practices in several different locations: the consulting room, the laboratory and the injection room. In this study, trained assessors spent three consecutive hours in each of 958 health-care facilities. The observational tool was used to record all interactions between patients and at least one health-care worker at each location. In collaboration with the Kenyan Ministry of Health and local experts, we identified three key procedures for observation: (i) physical examination; (ii) injection; and (iii) blood sampling. Five infection prevention and control domains were observed across these procedures: (i) hand hygiene; (ii) use of protective gloves; (iii) injection and blood sampling safety; (iv) disinfection of reusable equipment; and (v) waste segregation. These domains have been identified as critical for outpatient safety by WHO,^19^ the United States Centers for Disease Control and Prevention,^20^ the WHO Regional Committee for Africa^21^ and the Kenyan Ministry of Health.^22^ In accordance with the broad consensus that safety of care is a characteristic of the system and not just of individual health-care providers,^23^ for each of these domains, we assessed: (i) compliance with infection prevention and control practices by health-care workers; (ii) knowledge of these practices among health-care workers; and (iii) the availability of the equipment and supplies needed for implementing these practices.

### Assessing compliance

The assessment of compliance with infection prevention and control practices was based on indications and corresponding actions. An indication refers to a situation in which an infection prevention and control practice must be undertaken to prevent the risk of a pathogen being transmitted from one surface to another. Actions occur in response to indications, such that each indication has a corresponding action. Compliance means that the correct action has been taken. For example, for the domain of hand hygiene (Table 1), the indication “Before touching the patient” indicates the possibility that physical contact could lead to microbial transmission. The correct action corresponding to this indication is: “Health-care worker washed his or her hands with soap or used an alcohol-based hand rub.”^24^^–^^26^ We determined the proportion of indications that were accompanied by the corresponding action for all health-care workers across each of the 20 indications listed in Table 1 for five infection prevention and control domains. For example, for the domain of hand hygiene, compliance with seven well-known indications was assessed. Table 1 also lists the equipment and supplies essential for carrying out each action and Table 2 describes how health-care workers’ knowledge of the actions required in each domain was assessed. The novelty of our approach is that we used a single observational tool to collect comprehensive data on indications and their corresponding actions across a large number of infection prevention and control practices in an outpatient setting in a low-income country.^20^^,^^27^^,^^28^

**Table 1:** Indications for infection prevention and control practices and the action, equipment and supplies required for compliance, Kenya, 2015

Infection prevention and control domain and indication^a^	Corresponding action for compliance	Equipment and supplies required
**Hand hygiene**		
1. Before touching the patient	Health-care worker washed his or her hands with soap or used an alcohol-based hand rub	Running water^b^ and soap^c^ or an alcohol-based hand rub was available
2. After touching the patient	Health-care worker washed his or her hands with soap or used an alcohol-based hand rub	Running water^b^ and soap^c^ or an alcohol-based hand rub was available
3. Before a clean or aseptic procedure	Health-care worker washed his or her hands with soap or used an alcohol-based hand rub	Running water^b^ and soap^c^ or an alcohol-based hand rub was available
4. After exposure to body fluids	Health-care worker washed his or her hands with soap or used an alcohol-based hand rub	Running water^b^ and soap^c^ or an alcohol-based hand rub was available
5. After contact with an object that has touched the patient or was in the patient’s immediate environment	Health-care worker washed his or her hands with soap or used an alcohol-based hand rub	Running water^b^ and soap^c^ or an alcohol-based hand rub was available
6. Before an injection or taking a blood sample	Health-care worker washed his or her hands with soap or used an alcohol-based hand rub	Running water^b^ and soap^c^ or an alcohol-based hand rub was available
7. After an injection or taking a blood sample	Health-care worker washed his or her hands with soap or used an alcohol-based hand rub	Running water^b^ and soap^c^ or an alcohol-based hand rub was available
**Protective gloves**		
8. After using gloves with a patient^d^	Health-care worker used new gloves for each patient if gloves were used	Gloves were readily available
9. After using gloves in general^e^	Health-care worker washed his or her hands with soap or used an alcohol-based hand rub for each patient seen	Running water^b^ and soap^c^ or an alcohol-based hand rub was available
10. Before contact with blood, body fluids, mucous membranes, non-intact skin or contaminated equipment^f^	Health-care worker wore gloves	Gloves were readily available
11. After contact with blood, body fluids, mucous membranes, non-intact skin or contaminated equipment^f^	The health-care worker discarded the gloves into a waste bin	A waste bin was available
**Injections and blood samples**		
12. Before an injection or taking a blood sample	The health-care worker used a new needle	New needles were available^g^
13. Before an injection or taking a blood sample that required a syringe	The health-care worker used a new syringe	New syringes were available^g^
14. Before potential contact with blood, body fluids, mucous membranes, non-intact skin or contaminated equipment^f^	The health-care worker used clean swabs and alcohol-based solutions and did not use wet swabs from a multiuse container	ND
**Reusable equipment**		
15. Before or after patient contact (thermometer)	The health-care worker disinfected the thermometer using rubbing alcohol or bleach	Disinfectant or bleach was readily available^h^
16. Before or after patient contact (stethoscope)	The health-care worker disinfected the stethoscope using rubbing alcohol or bleach	Disinfectant or bleach was readily available^h^
**Waste segregation of needles and syringes**		
17. After an injection or taking a blood sample that required a syringe	The health-care worker segregated the syringe into a leak-proof, puncture-resistant sharps container	A leak-proof, puncture-resistant sharps container was readily available
18. After an injection or taking a blood sample in general	The health-care worker segregated the needle into a leak-proof, puncture-resistant sharps container	A leak-proof, puncture-resistant sharps container was readily available
**Waste segregation, excluding needles and syringes**		
19. After an injection or taking a blood sample during which infectious waste was produced	The health-care worker segregated swabs, gauzes and other infectious waste into a yellow or red bin with matching bag^i^	A yellow bin with a matching yellow bag or a red bin with a matching red bag was readily available
20. After a medical examination during which infectious waste was produced	The health-care worker segregated tongue depressors, swabs, gauzes and other infectious waste into a yellow or red bin with matching bag^j^	A yellow bin with a matching yellow bag or a red bin with a matching red bag was readily available

ND: not determined.

^a^ An indication refers to a situation in which an infection prevention and control practice must be undertaken to prevent the risk of a pathogen being transmitted from one surface to another.

^b^ Running water in a sink or from a bucket with a tap or a bucket with a pitcher.

^c^ Either bar or liquid soap.

^d^ This indication refers to situations in which a health-care worker reused gloves for successive patients, irrespective of whether glove use was recommended, and thereby created a risk that pathogens would be transferred.

^e^ This indication refers to situations in which a health-care worker removed his or her gloves, irrespective of the number of patient interactions or the type of procedure.

^f^ This definition is intended to cover injections and blood sampling.

^g^ Since the observational patient-tracking tool indicated that compliance with the use of new needles and syringes for injections and taking blood samples was 100%, it was concluded that new needles and syringes were available at all health-care facilities.

^h^ The availability of disinfectant or bleach was determined by an additional survey conducted by the research team on the same day as the study survey using a checklist on patient safety standards developed by the Kenyan Ministry of Health and medical boards and councils.

^i^ If a yellow or red bin with matching bag was not available, the health-care worker was regarded as not having complied with this practice.

^j^ A swab or gauze had to be segregated only if it had been used in an invasive patient contact or for a clean or aseptic procedure or had been exposed to body fluids. If a yellow or red bin with matching bag was not available, the health-care worker was regarded as not having complied with this practice.

**Table 2:** Assessing health-care workers’ knowledge of infection prevention and control practices, Kenya, 2015

Infection prevention and control domain and indication^a^	Question	Correct response^b^
**Hand hygiene**		
1. Before touching the patient 2. After touching the patient 3. Before a clean or aseptic procedure 4. After exposure to body fluids 5. After contact with an object that has touched the patient or is in the patient’s immediate environment 6. Before an injection or taking a blood sample 7. After an injection or taking a blood sample	Can you name the most important indications where hand hygiene is recommended during an interaction with a patient?	Study assessors were provided with a list of indications and were trained to classify health-care workers’ responses according to seven categories: (i) before touching a patient (indication 1); (ii) after touching a patient (indication 2); (iii) before a clean or aseptic procedure (indication 3); (iv) after exposure to body fluids (indication 4); (v) after contact with an object that has touched the patient or is in the patient’s immediate environment (indication 5); (vi) for indication 6, a response classified as either (i) or (iii) was sufficient; and (vii) for indication 7, a response classified as either (ii) or (iv) was sufficient
**Protective gloves**		
8. After using gloves with a patient^c^	Do you agree or disagree with the following statement: “Gloves can be used for more than one patient as long as they have not been exposed to blood or other body fluids”?	Disagree
9. After using gloves in general^d^	Do you agree or disagree with the following statement: “When using gloves, washing hands is not necessary after examining a patient”?	Disagree
10. Before contact with blood, body fluids, mucous membranes, non-intact skin or contaminated equipment^e^	Can you name the most important indications where wearing gloves is recommended in a health-care facility?	Study assessors were provided with a list of indications and were trained to classify health-care workers’ responses according to the following four correct indications: (i) to prevent contact with blood; (ii) to prevent contact with body fluids, mucous membranes or broken skin; (iii) before performing invasive medical procedures; and (iv) before touching a contaminated surface or contaminated waste
11. After contact with blood, body fluids, mucous membranes, non-intact skin or contaminated equipment^e^	Do you agree or disagree with the following statement: “Gloves should always be removed before leaving the area where the patient was seen”?	Agree
**Injections and blood samples**		
12. Before an injection or taking a blood sample	Do you agree or disagree with the following statement: “Needles should be used for only one patient”?	Agree
13. Before an injection or taking a blood sample that required a syringe	Do you agree or disagree with the following statement: “Syringes can be reused on more than one patient since they do not come into contact with the patient's body fluids”?	Disagree
14. Before potential contact with blood, body fluids, mucous membranes, non-intact skin or contaminated equipment^e^	ND	ND
**Reusable equipment**		
15. Before or after patient contact (thermometer)	Can you tell me when it is recommended to disinfect a thermometer?	After the thermometer was in contact with a patient
16. Before or after patient contact (stethoscope)	Can you tell me when it is recommended to disinfect a stethoscope?	After the stethoscope was in contact with a patient
**Waste segregation of needles and syringes**		
17. After an injection or taking a blood sample that required a syringe	Can you name the recommended type of container for segregating used syringes?	Study assessors were trained to classify health-care workers’ responses according to the following categories: (i) containers for highly infectious waste; (ii) containers for infectious or hazardous health-care waste; and (iii) containers for non-infectious waste
18. After an injection or taking a blood sample in general	Can you name the recommended type of container for segregating used needles?	Study assessors were trained to classify health-care workers’ responses according to the following categories: (i) containers for highly infectious waste; (ii) containers for infectious or hazardous health-care waste; and (iii) containers for non-infectious waste
**Waste segregation, excluding needles and syringes**		
19. After an injection or taking a blood sample during which infectious waste was produced 20. After a medical examination during which infectious waste was produced	Can you tell me what type of waste goes into each of the following colour-coded bins: red, yellow and black?	Red for highly infectious waste, yellow for infectious or hazardous health-care waste and black for non-infectious waste

ND: not determined.

^a^ An indication refers to a situation in which an infection prevention and control practice must be undertaken to prevent the risk of a pathogen being transmitted from one surface to another.

^b^ All responses were unprompted, except where the health-care worker was asked to agree or disagree with a statement.

^c^ This indication refers to situations in which a health-care worker reused gloves for successive patients, irrespective of whether glove use was recommended, and thereby created a risk that pathogens would be transferred.

^d^ This indication refers to situations in which a health-care worker removed his or her gloves, irrespective of the number of patient interactions or the type of procedure.

^e^ This definition is intended to cover injections and blood sampling.

A strict survey protocol ensured that study assessors did not interact with health-care workers or patients during observation. Data quality was assured using several methods: (i) the assessors’ skills were evaluated using tests and videos; (ii) data forms were reviewed daily; (iii) inter-rater reliability was evaluated on a sample of observations; and (iv) data were double-entered to ensure an error rate below 1%. Data collected by the observational, patient-tracking tool were recorded on paper and tablet computers were used in assessing the availability of supplies and health-care workers’ knowledge.

### Health-care facilities

The observational tool was piloted in 605 patients at 23 health-care facilities in Nairobi, which were selected to represent the wide range of facilities that provide primary health care in Kenya. They included public, private not-for-profit and private commercial facilities of differing levels of complexity: level-2 facilities included basic dispensaries and clinics, whereas level-5 facilities included county referral hospitals offering both basic and specialized services.

The main study took place in Kakamega, Meru and Kilifi counties in different regions of Kenya. Consent was obtained from 1035 of the 1115 (93%) health-care facilities identified and 935 took part in the study: 94 had no patients on the survey day and 6 did not provide consent on that day. In addition, 99% (14 443/14 531) of patients and 100% (1680/1680) of health-care workers approached also consented to being observed. The characteristics of participating facilities, health-care workers and patients are listed in Table 3. Although the distribution of facilities by level of complexity was similar to that at the national and county level, the proportion of private facilities was higher, possibly because these facilities were under-represented in administrative data.

**Table 3:** Health-care facilities, health-care workers and patients, infection prevention and control study, Kenya, 2015

Characteristic	No. (%)^a^
**Health-care facilities**	935 (100)
Public	369 (39)
Private	566 (61)
Level 2^b^	766 (82)
Level 3^b^	121 (13)
Level 4 or 5^b^	48 (5)
Number of outpatients seen per month, mean (SD)	631 (973)
**Health-care workers^c^**	1 636 (100)
Male	834 (51)
Age, years	
19 to 30	550 (34)
31 to 55	867 (53)
> 55	219 (13)
Highest educational level achieved	
Primary or secondary school	65 (4)
College certificate	458 (28)
College diploma	998 (61)
Bachelor’s, master’s or doctoral degree	115 (7)
Days worked at this facility each week, mean (SD)	5.43 (0.91)
**Patients^d^**	14 328 (100)
Male	5 664 (40)
Age, years	
< 5	3 862 (27)
5 to 18	3 579 (25)
19 to 30	2 562 (18)
31 to 55	3 204 (22)
> 55	1030 (7)
**Health-care worker–patient interactions completed**	18 826 (100)
Length of interactions in minutes, mean (SD)	4.55 (4.74)
**Procedures observed by study assessors^e^**	21 791 (100)
Examinations	14 300 (66)
Injections	2 451 (11)
Blood sampling	5 040 (23)
**Infection prevention and control indications observed^f^**	106 464 (100)

SD: standard deviation.

^a^ All values represent absolute numbers and percentages unless otherwise stated.

^b^ Level-2 facilities included dispensaries and clinics, level-3 facilities included health centres and maternity and nursing homes, level-4 facilities included primary hospitals (i.e. hospitals with the capacity to carry out emergency surgery, at least, and with a catchment area of at least 100 000 people) and level-5 facilities included secondary hospitals (i.e. hospitals that provided a higher level of specialized services and clinical supervision, supported primary referral facilities and had a catchment area of at least 1 000 000 people).

^c^ We could not interview 44 of the 1680 health-care workers observed.

^d^ Data on gender and age were missing for 133 and 91 patients, respectively, of the 14 328 observed.

^e^ Interactions between health-care workers and patients can involve several procedures, such as an examination and an injection or an injection and blood sampling. In addition, patients may interact with more than one health-care worker.

^f^ An indication refers to a situation in which an infection prevention and control practice must be undertaken to prevent the risk of a pathogen being transmitted from one surface to another (Table 1).

Overall, we observed 21 791 procedures in the main study (i.e. physical examination, injection or blood sampling) performed by 1680 health-care workers in 14 328 patients and we registered 106 464 indications for infection prevention and control practices. In higher-level facilities (i.e. levels 3, 4 and 5) with two or more health-care workers, we observed each worker for at least 1 hour. The mean age of the health-care workers was 37.4 years, they worked a mean of 5.43 days a week in their facilities and 51% were male (Table 3). Of the patients, 40% (5664/14 328) were male and 52% (7441/14 328) were younger than 18 years. Because public facilities see more patients, 70% (9976/14 328) were observed at public facilities even though only 39% (369/935) of facilities were public. 

The study was approved by the Ethics and Scientific Review Board at the African Medical and Research Foundation (Approval no. AMREF-ESRC P94/2013), the Kenyan Ministry of Health and authorities at participating facilities.

### Statistical analysis

We calculated the compliance by dividing the number of correct actions performed by the total number of indications and report the observed compliance with infection prevention and control practices for each domain (i.e. aggregated across indications) and for all domains combined. This implies that the implicit weight given to each individual action is the frequency with which it was observed. We adopted this approach because the relative risk associated with different infection prevention and control practices in primary health care has not been established. To assess whether compliance was associated with the individual characteristics of the health-care worker or facility, we proceeded in a stepwise fashion. First, we used multiple linear regression to assess the association between the availability of supplies and compliance. Then, we used a similar analysis to assess the association between other characteristics and compliance in the subset of observations for which supplies were available since the compliance would necessarily be zero if essential supplies were unavailable. In particular, it can be shown that, when supplies are necessary but not sufficient for compliance, the lower bound for the association between the availability of supplies and compliance is the mean compliance in the sample. Consequently, the regression coefficient for the association between the availability of supplies and compliance will lie between the mean compliance in the sample and 1. In deriving standard errors, we adjusted for the effect of clustering at the level of the facility and of the health-care worker. Data were analysed using Stata version 13.0 (StataCorp. LP, College Station, United States of America). Further details of the statistical methods are available from the corresponding author.

## Results

In the pilot study, we found that: (i) the level of consent was high, with 98% (605/617) of patients and 98% (50/51) of health-care workers consenting; (ii) the use of identification tags to track patients across different units in a facility was effective; and (iii) inter-rater agreement on assessments was high (kappa: 0.72; 95% confidence interval, CI: 0.68 to 0.76), with 93% of comparisons in agreement. The mean overall compliance across the five infection prevention and control domains was 0.382 (95% CI: 0.366 to 0.399) and the mean number of safety violations per patient was 3.7 (95% CI: 3.6 to 3.8). The mean compliance was highest for the injections and blood samples domain, at 0.988 (95% CI: 0.980 to 0.996), and lowest for hand hygiene, at 0.028 (95% CI: 0.020 to 0.037).

**Fig. 1:**
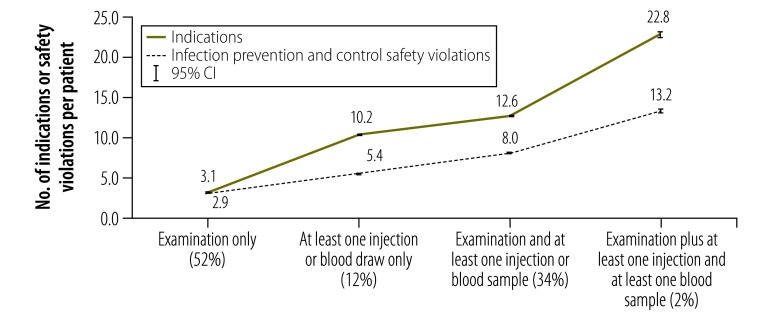
Infection prevention and control indications and safety violations, infection prevention and control study, Kenya, 2015

**Fig. 2:**
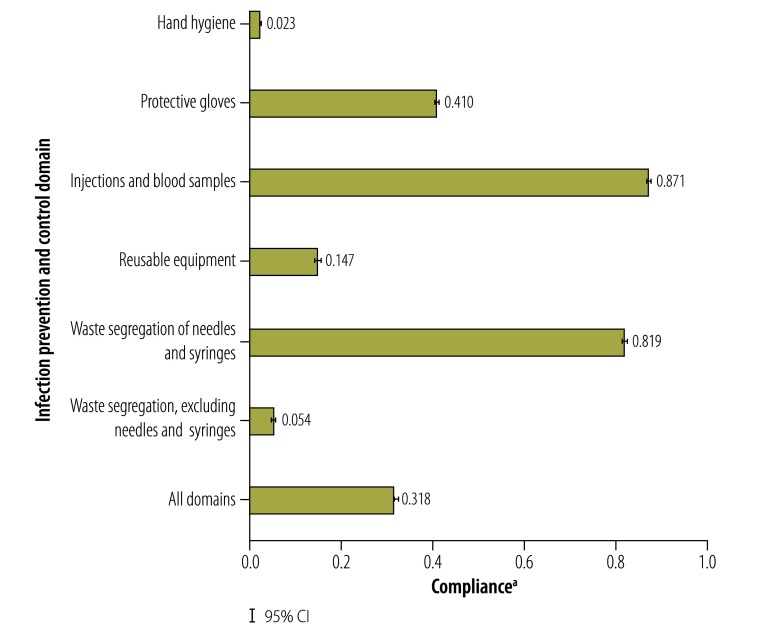
Compliance with infection prevention and control practices, by infection prevention and control domain, Kenya, 2015

In the main study, the mean number of infection prevention and control indications per patient was 7.5 (95% CI: 7.4 to 7.6) and the mean number of safety violations per patient was 5.1 (95% CI: 5.1 to 5.2) for each outpatient visit. The mean overall compliance was 0.318 (95% CI: 0.315 to 0.321) for the 106 464 indications observed. The number of indications and safety violations increased with the number of procedures but compliance varied according to the specific procedures performed (Fig. 1). This observation is consistent with the substantial variation in compliance across domains (Fig. 2). For example, the mean compliance in the injections and blood samples domain was 0.871 (95% CI: 0.866 to 0.876) compared with 0.023 (95% CI: 0.021 to 0.024) in the hand hygiene domain.

CI: confidence interval.

Notes: An indication refers to a situation in which an infection prevention and control practice must be undertaken to prevent the risk of a pathogen being transmitted from one surface to another (Table 1). A safety violation occurred when the required action was not taken. The percentages in parenthesis give the proportion of patients who underwent the procedure or combination of procedures.

CI: confidence interval.

^a^ The compliance is the proportion of indications for an infection prevention and control practice for which the corresponding action was taken.

Notes: An indication refers to a situation in which an infection prevention and control practice must be undertaken to prevent the risk of a pathogen being transmitted from one surface to another (Table 1). The data in the figure relate to all 106 464 indications observed.

We found small, weak associations between compliance and most characteristics of health-care workers and facilities. The proportion of indications for which the health-care worker had the requisite knowledge and the proportion for which the requisite supplies were available were considerably higher than the rate of compliance for those indications in most domains (Table 4). In the hand hygiene domain, the mean compliance was 0.042 when the health-care worker had the requisite knowledge and the requisite supplies compared with an overall mean of 0.024. The regression coefficient for the association between compliance and the availability of supplies determined using the ordinary least squares method is 0.368 (95% CI: 0.352 to 0.385; Table 5), which was very close to the observed overall mean compliance of 0.318. However, after domain fixed effects were taken into account, the regression coefficient was 0.162 (95% CI: 0.145 to 0.179), which suggests that supplies were necessary but not sufficient for compliance.

**Table 4:** Health-care workers’ knowledge, availability of supplies and compliance with infection prevention and control practices, Kenya, 2015

Infection prevention and control domain	All indications,^a^ no.	Proportion of indications for which the health-care worker had the requisite knowledge, mean (SE)^c^	Proportion of indications for which the requisite supplies were available, mean (SE)^c^	Compliance,^b^ mean (SE)^c^	Indications for which the health-care worker had the requisite knowledge, mean (SE)^c^	Compliance,^b^ mean (SE)^c^	No. of indications for which the health-care worker had the requisite knowledge and the requisite supplies were available	Compliance,^b^ mean (SE)^c^
Hand hygiene	41 267	0.517 (0.002)	0.704 (0.002)	0.024 (0.001)	21 327	0.032 (0.001)	15 079	0.042 (0.002)
Protective gloves	18 517	0.907 (0.002)	0.848 (0.003)	0.412 (0.004)	16 802	0.424 (0.004)	14 248	0.487 (0.004)
Injections and blood samples	9 064	0.993 (0.001)	1.000 (0.000)	0.997 (0.001)	8 998	0.997 (0.001)	8 998	0.997 (0.001)
Reusable equipment	2 581	0.878 (0.006)	0.636 (0.009)	0.148 (0.007)	2 266	0.165 (0.008)	1 437	0.165 (0.010)
Waste segregation of needles and syringes	8 966	0.939 (0.003)	0.867 (0.004)	0.822 (0.004)	8 420	0.847 (0.004)	7 459	0.942 (0.003)
Waste segregation, excluding needles and syringes	8 419	0.718 (0.005)	0.267 (0.005)	0.058 (0.003)	6 043	0.069 (0.003)	1 917	0.217 (0.009)
**All domains**	**88 814^d^**	**0.719 (0.002)**	**0.738 (0.001)**	**0.292 (0.002)**	**63 856**	0.387 (0.002)	49 138	0.493 (0.002)

SE: standard error.

^a^ An indication refers to a situation in which an infection prevention and control practice must be undertaken to prevent the risk of a pathogen being transmitted from one surface to another.

^b^ The compliance is the proportion of indications for an infection prevention and control practice for which the corresponding action was taken (Table 1).

^c^ Robust SE (i.e. robust to heteroscedasticity or unequal variances).

^d^ Data on health-care workers’ knowledge and supplies were available for 88 814 of the 106 464 indications for which data on compliance were recorded.

**Table 5:** Association between compliance with infection prevention and control practices and health-care workers’ and facilities characteristics, Kenya, 2015^a ^

Variable	Regression coefficient estimate,^b,c^ mean (SE)^d^				
	All indications^e^ (*n* = 92 430)^f^		Indications^e^ for which the requisite supplies were available (*n* = 64 494)^g^		
	Ordinary least squares^h^	Domain fixed-effects^i^	Ordinary least squares^h^	Domain fixed effects^i^	Health-care worker fixed effects^i^
**Availability of supplies required for infection prevention and control practices**	0.368 (0.008)***	0.162 (0.009)***	N/A	N/A	N/A
**Health-care workers’ knowledge of infection prevention and control practices**	ND	ND	0.412 (0.010)***	0.035 (0.007)***	0.026 (0.008)***
**Kenyan infection prevention and control guidelines available at the facility**	ND	ND	0.045 (0.021)**	0.012 (0.015)	ND
**Health-care workers underwent training on infection prevention and control within the last calendar year**	ND	ND	−0.016 (0.017)	0.009 (0.011)	ND
**Health-care workers’ highest educational level^j^**					
College diploma	ND	ND	−0.130 (0.018)***	−0.018 (0.010)*	ND
Bachelor’s, master’s or doctoral degree	ND	ND	−0.133 (0.029)***	−0.026 (0.014)*	ND
**Age of health-care worker, per year**	ND	ND	−0.003 (0.001)***	−0.001 (0.000)**	ND
**Male health-care worker**	ND	ND	0.002 (0.015)	−0.019 (0.008)**	ND
**Public health facility**	ND	ND	−0.005 (0.014)	−0.006 (0.008)	ND
**Health-care facility level^k^**					
Level 3	ND	ND	0.016 (0.020)	−0.003 (0.010)	ND
Level 4	ND	ND	0.013 (0.025)	0.004 (0.014)	ND
Level 5	ND	ND	0.063 (0.065)	0.007 (0.020)	ND
**Order number of patients observed**	ND	ND	−0.001 (0.001)**	−0.001 (0.000)**	−0.000 (0.000)
**Infection prevention and control domain indicators^l^**					
Hand hygiene	ND	−0.923 (0.005)***	ND	−0.942 (0.006)***	−0.944 (0.008)***
Protective gloves	ND	−0.492 (0.013)***	ND	−0.446 (0.015)***	−0.475 (0.015)***
Reusable equipment	ND	−0.790 (0.017)***	ND	−0.843 (0.022)***	−0.818 (0.025)***
Waste segregation of needles and syringes	ND	−0.155 (0.013)***	ND	−0.061 (0.009)***	−0.060 (0.009)***
Waste segregation, excluding needles and syringes	ND	−0.820 (0.012)***	ND	−0.769 (0.030)***	−0.773 (0.034)***
**Adjusted *R^2^***	0.127	0.633	0.154	0.650	0.588

N/A: not applicable; ND: not determined; *: *P* < 0.1; **: *P* < 0.5; ***: *P* < 0.01; SE: standard error.

^a^ Further details of the statistical analysis, including the robustness analysis for missing observations, are available from the corresponding author.

^b^ All values in the table represent regression coefficients unless otherwise stated.

^c^ Regression coefficients for the association between compliance with infection prevention and control practices and explanatory variables as determined by five linear multiple regression specifications. All models included a constant (coefficient estimates excluded). The third and fourth models included county dummies (coefficient estimates excluded).

^d ^Robust SEs are clustered at the health facility level to account for clustering and heteroscedasticity or unequal variances.

^e^ An indication refers to a situation in which an infection prevention and control practice must be undertaken to prevent the risk of a pathogen being transmitted from one surface to another (Table 1).

^f^ Data on the availability of supplies were recorded for 92 430 of the 106 464 Infection prevention and control indications observed.

^g^ Data on health-care workers’ knowledge and other characteristics were recorded for 64 494 observed indications for which supplies were available. Data were missing because: (i) some health-care workers were not interviewed due to very high patient caseloads; and (ii) information on knowledge and supplies were not collected for one indication (i.e. 14. Before potential contact with blood, body fluids, mucous membranes, non-intact skin or contaminated equipment; Table 1).

^h^ The regression analysis was based on the ordinary least squares method.

^i^ The regression analysis took into account domain fixed effects for infection prevention and control domains and health-care worker fixed effects, as appropriate.

^j^ College certificate level and below were excluded.

^k^ Level 2 was excluded.

^l^ The injections and blood samples domain was excluded.

For the 68 034 observed indications for which supplies were available, the average compliance was 0.390 (95% CI: 0.386 to 0.393). Table 5 lists regression coefficients for the association between compliance and other health-care worker and facility characteristics in the subset of 64 494 observations for which supplies were available and data on health-care workers’ knowledge were recorded. Regression coefficients were derived after separately taking into account domain fixed effects and adding health-care worker fixed effects, which enabled us to assess whether differences in the supplies available or in knowledge between individual health-care workers were associated with compliance. In this subset of observations, the estimated regression coefficients for compliance, after domain fixed effects were taken into account, were most strongly associated with the domain – the inclusion of indicator variables for the domains increased the adjusted *R^2^* (which indicates how close data are to the fitted regression line) for the regression from 0.154 to 0.650. In contrast, compliance was weakly associated with the type of facility (i.e. public or private or level of specialization), the health-care worker’s educational level, age and sex, the availability of Kenyan infection prevention and control guidelines (only 5% of facilities had a copy) and whether the health-care worker had undergone training on infection prevention and control within the last year. Also in the subset of observations for which supplies were available, the regression coefficient for the association between compliance and health-care workers’ knowledge was 0.035 (95% CI: 0.021 to 0.050) after domain fixed effects were taken into account and 0.026 (95% CI: 0.011 to 0.041) once health-care worker fixed effects (e.g. an individual worker’s level of motivation) were taken into account.

Table 6 shows the association between compliance and the availability of supplies separately for each infection prevention and control domain: the regression coefficient for compliance ranged from 0.006 (95% CI: −0.059 to 0.071) for the reusable equipment domain to 0.848 (95% CI: 0.801 to 0.894) for the waste segregation of needles and syringes domain. Regression coefficients for the association between compliance and health-care workers’ knowledge when supplies were available (Table 7) were smaller and few were statistically significant. The largest coefficients were 0.184 (95% CI: 0.066 to 0.302) for the waste segregation of needles and syringes domain and 0.151 (95% CI: 0.083 to 0.219) for the reusable equipment domain.

**Table 6:** Association between compliance with infection prevention and control practices and the availability of supplies, by infection prevention and control domain, Kenya, 2015^a^

Variable	Infection prevention and control domain					
	Hand hygiene	Protective gloves	Injections and blood samples	Reusable equipment	Waste segregation of needles and syringes	Waste segregation, excluding needles and syringes
No. of indications^a^	46 006	15 967	9499	2768	9389	8801
Estimated regression coefficient for the association between compliance with infection prevention and control practices and the availability of supplies,^b^ mean (SE)^c^	0.028 (0.004)***	0.454 (0.023)***	ND^d^	0.006 (0.033)	0.848 (0.024)***	0.219 (0.029)***
Constant, mean (SE)^c^	0.006 (0.001)***	0.089 (0.018)***	0.997 (0.001)***	0.145 (0.026)***	0.087 (0.022)***	−0.000 (0.000)
Adjusted *R^2^*	0.007	0.094	0.000	−0.000	0.568	0.171

ND: not determined; ***: *P* < 0.01; SE: standard error.

^a^ An indication refers to a situation in which an infection prevention and control practice must be undertaken to prevent the risk of a pathogen being transmitted from one surface to another (Table 1).

^b^ Regression coefficients were determined by linear multiple regression.

^c^ Standard errors are clustered at the health facility level.

^d^ The regression coefficient was not estimated for the injections and blood samples domain because supplies were always available.

**Table 7:** Association between compliance with infection prevention and control practices and health-care workers’ and facilities characteristics when supplies were available, by infection prevention and control domain, Kenya, 2015^a^

Variable	Regression coefficient estimate,^b^ mean (SE),^c,d^ per infection prevention and control domain					
	Hand hygiene	Protective gloves	Injections and blood samples	Reusable equipment	Waste segregation of needles and syringes	Waste segregation, excluding needles and syringes
**Health-care workers’ knowledge of infection prevention and control practices**	0.023 (0.005)***	0.062 (0.048)	−0.003 (0.003)	0.151 (0.035)***	0.184 (0.060)***	0.030 (0.064)
**Kenyan infection prevention and control guidelines available at the facility**	0.005 (0.010)	0.050 (0.049)	0.002 (0.002)	−0.086 (0.048)*	0.006 (0.030)	0.035 (0.070)
**Health-care workers underwent training on infection prevention and control within the last calendar year**	0.017 (0.012)	0.028 (0.032)	−0.003 (0.003)	−0.022 (0.033)	−0.031 (0.022)	−0.022 (0.055)
**Health-care workers’ highest educational level^e^**						
College diploma	−0.004 (0.008)	−0.070 (0.032)**	0.003 (0.002)	−0.005 (0.047)	−0.008 (0.019)	−0.089 (0.054)*
Bachelor’s, master’s or doctoral degree	0.005 (0.013)	−0.090 (0.048)*	−0.000 (0.003)	0.003 (0.071)	−0.071 (0.046)	−0.067 (0.094)
**Age of health-care worker, per year**	−0.000 (0.000)	−0.003 (0.001)*	−0.000 (0.000)	−0.001 (0.001)	−0.000 (0.001)	−0.002 (0.003)
**Male health-care worker**	−0.007 (0.008)	−0.056 (0.028)**	−0.001 (0.002)	−0.016 (0.038)	−0.015 (0.017)	−0.052 (0.050)
**Public health facility**	−0.014 (0.008)*	−0.032 (0.028)	0.005 (0.003)*	0.082 (0.035)**	0.045 (0.019)**	−0.061 (0.064)
**Health-care facility level^f^**						
Level 3	−0.022 (0.008)***	0.030 (0.034)	0.003 (0.001)***	−0.051 (0.081)	0.027 (0.018)	−0.028 (0.066)
Level 4	−0.032 (0.011)***	0.057 (0.041)	0.001 (0.002)	−0.084 (0.063)	0.016 (0.039)	0.117 (0.069)*
Level 5	−0.031 (0.018)*	−0.063 (0.048)	0.002 (0.002)	−0.181 (0.049)***	0.062 (0.037)*	0.243 (0.116)**
**Order number of patients observed**	−0.000 (0.000)	−0.004 (0.001)***	−0.000 (0.000)	0.002 (0.002)	0.001 (0.000)	0.001 (0.001)
**No. of indications^g^**	30 580	13 265	9027	1637	7739	2246
**Adjusted *R^2^***	0.015	0.027	0.006	0.058	0.051	0.036

*:*P* < 0.1; **: *P* < 0.5; ***: *P* < 0.01; SE: standard error.

^a^ Further details of the statistical analysis are available from the corresponding author on request.

^b^ Regression coefficients for the association between compliance with infection prevention and control practices and explanatory variables as determined by linear multiple regression.

^c^ Standard errors are clustered at the health facility level.

^d^ All values in the table are means and standard errors unless otherwise stated.

^e^ College certificate level and below were excluded.

^f^ Level 2 was excluded.

^g^ An indication refers to a situation in which an infection prevention and control practice must be undertaken to prevent the risk of a pathogen being transmitted from one surface to another (Table 1).

In domains, such as the hand hygiene domain, where the mean compliance and estimated regression coefficients for the association between compliance and the availability of supplies and knowledge were all small, it is unlikely that a lack of supplies or knowledge was the only constraint. In contrast, in domains such as the waste segregation excluding needles and syringes domain, where the regression coefficient for the association between compliance and the availability of supplies was higher and the observed compliance was small, it is likely that the availability of supplies was a more important constraining factor.

## Discussion

Our observational, patient-tracking tool was able to assess compliance with infection prevention and control practices across a range of health-care facilities and infection prevention and control domains. We found there were, on average, 13 opportunities for infection transmission during an outpatient visit if the patient was examined, had an injection and underwent blood sampling. Compliance varied widely across indications: it was almost complete for single-use needles and syringes but very low for hand hygiene – when practiced, hand washing lasted an average of 16 s compared with the recommended 30 to 60 s. Data collected using the tool can provide key information for epidemiological modelling of disease outbreaks because different safety violations may be associated with different risks depending on the disease transmission mechanism.^29^^,^^30^

We found only weak associations between compliance and the availability of supplies, health-care workers’ knowledge, training in infection prevention and control and the availability of guidelines. These findings are consistent with the widely discussed concept that patient safety is driven more by behavioural norms than by technical knowledge, training or the availability of supplies.^12^^,^^31^ Consequently, compliance depends on engendering these norms, which has been achieved in Kenya for injections and blood sampling but not for hand hygiene. There has been substantial decline in unsafe injection practices worldwide,^32^ which could be extended to other practices. In Australia, for example, altering behavioural norms in hospitals has substantially improved hand hygiene.^33^

Our observational tool for assessing infection prevention and control practices across multiple domains has several limitations. First, linking practices to health outcomes requires data on the types of pathogens present at observation sites – this would ultimately enable researchers to apply an appropriate weighting to compliance with specific infection prevention and control indications. However, there are no literature reports on the relative risks of different practices, even in high-income countries. Moreover, although WHO suggests that it is reasonable to focus on key domains that are consistently linked with nosocomial infections (e.g. hand hygiene is considered to be the single most effective infection control measure),^1^ the evidence from outpatient settings is sparse. Second, our tool was based on direct observations, which could have been subject to the Hawthorne effect, whereby health-care providers changed their behaviour when observed. However, previous studies of this effect suggest that observation-induced behaviour decreases with the number of interactions observed.^34^ We examined whether early observations differed from later observations and, although we found a small, negative association, it disappeared once we controlled for the infection prevention and control domain and health-care worker fixed-effects. The absence of a Hawthorne effect is encouraging because some researchers consider direct observation to be the gold standard for measuring compliance with infection prevention and control practices since it makes it possible to record both indications and their corresponding actions.^20^^,^^25^ Finally, our data may be incomplete because they relate only to the day of observation. For example, waste disposal (not waste segregation) may take place on only one day of the week or month. Although we were unable to comprehensively analyse waste disposal, there was evidence of important gaps in waste management. For instance, only 11.1% of facilities had a standard operating procedure for waste management, only 26.1% had an on-site incinerator or a contract with a company for incineration and only 27.8% had a waste holding area.

In conclusion, our observational, patient-tracking tool provided an effective way of assessing compliance with infection prevention and control practices across multiple domains in primary health care. It could be used to rapidly assess the current status of these practices and to monitor improvement efforts. We found that compliance with infection prevention and control practices was low overall but varied substantially across domains. The variations were only weakly associated with the characteristics of the facility and the health-care worker, such as the health-care worker’s knowledge and the availability of supplies, which suggests that improvements will require a broader focus on behavioural change.
